# Preliminary evaluation of the TB-IGRA VIRCLIA® Monotest chemiluminescent interferon-gamma release assay: a comparative agreement study with QuantiFERON-TB Gold Plus

**DOI:** 10.3389/fmicb.2026.1916499

**Published:** 2026-10-29

**Authors:** Jose Mária Serrano, Maria Aguilera-Franco, Juan Francisco Gutiérrez-Bautista, Fernando Cobo, M. J. Olivares Duran, Antonio Sampedro-Padilla, F. Ruiz, Jose Gutiérrez Fernández, José María Navarro-Mari, Javier Rodriguez-Granger

**Affiliations:** 1 Microbiology Service, Virgen de las Nieves University Hospital, Granada, Spain; 2 Clinical Medicine and Public Health Doctoral Program, Graduate School, University of Granada, Granada, Spain; 3 Clinical Analysis and Immunology Service, Virgen de las Nieves University Hospital, Granada, Spain; 4 Institute Biosanitary of Granada (ibs.GRANADA), Granada, Spain; 5 Zaidin Sur Health Center, Andalusian Health Service, Granada, Spain; 6 Department Medical Centre Penitentiary, Granada, Spain; 7 Department of Microbiology, School of Medicine, University of Granada, Granada, Spain

**Keywords:** chemiluminescence, interferon-gamma release assay, QuantiFERON-TB Gold Plus, Tuberculosis infection, VIRCLIA

## Abstract

**Objectives:**

We evaluated the qualitative and quantitative agreement of the novel chemiluminescent TB-IGRA VIRCLIA® Monotest with QuantiFERON®-TB Gold Plus (QFT-Plus) for detecting tuberculosis infection.

**Methods:**

In this prospective comparative study, 226 consecutive samples submitted for routine tuberculosis infection testing were analyzed using the TB-IGRA VIRCLIA® Monotest and the QFT-Plus chemiluminescence immunoassay (CLIA). Agreement between the two tests was assessed by calculating the overall agreement, positive and negative percent agreement (PPA/NPA), Cohen’s kappa (*κ*), Deming regression, and Bland–Altman analysis. An exploratory QFT-Plus-derived borderline range (0.20–0.70 IU/mL) was analyzed separately. Additional quantitative analyses were performed after excluding observations at the QFT-Plus upper quantification limit (10 IU/mL).

**Results:**

Overall qualitative agreement was 92.9% (*κ* = 0.85; 95% confidence interval (CI), 0.79–0.92), with PPA of 90.1% (95% CI, 83.8–94.1) and NPA of 96.8% (95% CI, 91.1–98.9). Agreement was 94.25% for TB1 (*κ* = 0.885; 95% CI, 0.824–0.945) and 89.82% for TB2 (*κ* = 0.796; 95% CI, 0.718–0.875). In the borderline range, agreement decreased to 78.05% for TB1 (*κ* = 0.529; 95% CI, 0.289–0.769) and 69.05% for TB2 (*κ* = 0.316; 95% CI, 0.067–0.564), compared to 97.84 and 94.57%, respectively, for values outside this range. Deming regression slopes decreased from 3.085 to 0.811 for TB1 and from 3.146 to 0.926 for TB2 after excluding observations at the QFT-Plus upper quantification limit, with substantially narrower Bland–Altman limits of agreement.

**Conclusion:**

The TB-IGRA VIRCLIA® Monotest showed a high overall qualitative agreement with QFT-Plus, particularly outside the borderline range. Agreement decreased near the positivity threshold, particularly for TB2, supporting cautious interpretation and potential repeat or confirmatory testing of borderline results. Quantitative comparisons at high interferon-gamma (IFN-γ) concentrations were substantially influenced by the QFT-Plus upper quantification limit.

## Introduction

Tuberculosis (TB) remains a global health problem, with 10.7 million reported cases and 1.23 million deaths (Global Tuberculosis Report 2025, 2025). An even greater number of people, with estimates suggesting one-quarter of the global population, have tuberculosis infection (TBI), a condition in which individuals are infected with *Mycobacterium tuberculosis* but show no clinical symptoms (Hunter, 2016). One of the most effective TB preventive strategies is the treatment of high-risk individuals with *M. tuberculosis* infection. This is considered key in the control of TB in low-incidence TB settings (Collin et al., 2019). Accurate diagnosis of *M. tuberculosis* infection is crucial for effectively delivering preventive therapy. However, there is no definitive gold standard test for diagnosing TBI. To this end, the World Health Organization (WHO) recommends the use of either the tuberculin skin test (TST) or interferon-gamma release assays (IGRAs) [World Health Organization (WHO), 2022]. Traditionally, the diagnosis of TBI has been based on the TST. However, this test has well-known limitations, such as cross-reactivity with the bacille Calmette–Guérin (BCG) vaccine and non-tuberculous mycobacteria, as well as inter-observer variability that can affect its reproducibility (Gong and Wu, 2021; Gualano et al., 2019). In this context, IGRAs have emerged as a more specific alternative, as they utilize antigens specific to *M. tuberculosis* that are absent in the majority of environmental mycobacteria and in the BCG vaccine strain (Pai et al., 2014).

Despite their advantages, currently available IGRA tests present challenges relating to their cost, technical requirements, and accessibility, which may limit their routine implementation in certain clinical and epidemiological settings. Consequently, there is growing interest in developing new commercial kits that maintain adequate diagnostic performance while being simpler, standardized, and adaptable to different contexts of use (Li et al., 2023).

Until a few years ago, WHO recommendations for tuberculosis infection testing were limited to three commercial IGRAs: T-SPOT. TB (Oxford Immunotec, UK), QuantiFERON-TB Gold Plus (QFT-Plus; Qiagen, USA), and Wantai TB-IGRA (Wantai, China). More recently, these recommendations have been expanded to include additional assays, such as the STANDARD E TB-Feron enzyme-linked immunosorbent assay (ELISA) and the LIAISON® QuantiFERON®-TB Gold Plus chemiluminescence immunoassay (CLIA), broadening the range of available platforms for interferon-gamma (IFN-γ)-based tuberculosis infection testing (Li et al., 2023; World Health Organization, 2025).

Early studies assessing the diagnostic performance of IGRAs confirmed a higher specificity relative to the TST, although their sensitivity was similar (Pai et al., 2014; Menzies et al., 2007). For that reason, an IGRA assay such as the QuantiFERON Gold Plus test (QFT-Plus; Qiagen, Hilden, Germany) was launched in 2015 and added a new stimulation tube using the same Early Secreted Antigenic Target of 6 kDa (ESAT-6) and Culture Filtrate Protein of 10 kDa (CFP-10) antigens but designed to induce a specific Cluster of Differentiation 8 (CD8) response to increase its sensitivity (Qiagen, 2019). In 2021, a chemiluminescence immunoassay analyzer (Liaison XL) was adapted to the QFT-Plus to fully automate IFN-γ quantification (QFT-Plus CLIA, Qiagen/Diasorin) (De Maertelaere et al., 2020).

In addition, several commercially available assays based on interferon measurements are being launched or remain under development, employing different technological formats. In this context, QIAreach QuantiFERON-TB (QIAreach QFT, USA) and ichroma™ IGRA-TB (Boditech Med Inc., Korea) are lateral-flow immunochromatographic assays designed for rapid, point-of-care testing with simplified workflows. In contrast, ELISA-based platforms, including Standard E TB-Feron (SD Biosensor, Korea), LIOferon TB/LTBI (Lionex Diagnostic Therapeutics GmbH, Germany), and AdvanSure TB-IGRA ELISA (LG Life Sciences, Seoul, Korea), enable batch processing with established analytical performance. Furthermore, fully automated CLIA systems, such as VIDAS TB-IGRA (bioMérieux, France), integrate sample processing and detection, thereby improving standardization and reducing operator hands-on time (Goletti et al., 2025; Serge Diagbouga et al., 2025).

Overall, these assays show promising diagnostic agreement with established IGRAs and acceptable reproducibility, although most of these newer platforms still require further large-scale, multicenter clinical validation to confirm their reliability, reproducibility, and diagnostic accuracy across diverse epidemiological settings.

Several novel assays based on alternative strategies to classical IGRA are under development for the diagnosis of tuberculosis infection. For instance, T-Track® TB (Mikrogen GmbH, Germany) is based on the quantification of IFN-γ and C-X-C Motif Chemokine Ligand 10 (CXCL10), Interferon Gamma-Induced Protein 10 (IP-10) messenger RNA (mRNA) expression in antigen-stimulated whole blood using reverse transcription quantitative polymerase chain reaction (PCR). In addition, interferon protein-10 (IP-10) IGRA assays, available in ELISA and lateral-flow formats, measure the release of CXCL10 (IP-10), a chemokine induced by IFN-*γ*, as an alternative biomarker of *Mycobacterium tuberculosis*-specific immune activation (Madhurantakam et al., 2023; Saluzzo et al., 2022).

Laboratories can differ substantially in terms of infrastructure, technical capacity, and workload, while epidemiological factors such as TB prevalence and population risk profiles also influence diagnostic needs. Therefore, the availability of diverse IGRA formats is essential to ensure accurate and accessible diagnosis of tuberculosis infection across heterogeneous epidemiological settings. The TB-IGRA VIRCLIA® Monotest (Vircell S. L., Granada, Spain) is a chemiluminescent assay designed to quantify IFN-γ responses following stimulation in QFT-Plus Nil, Mitogen, TB1, and TB2 tubes. Its Monotest format allows automated processing of individual samples without the need to accumulate specimens for batch testing, which may be particularly useful in laboratories with low or irregular IGRA workloads. However, independent clinical data comparing this platform with established IGRA methods remain limited.

The aim of the present study was therefore to evaluate the qualitative and quantitative agreement between the TB-IGRA VIRCLIA® Monotest and QFT-Plus CLIA in consecutive samples submitted for routine TBI testing in a low-incidence setting in southern Spain. We additionally examined agreement around an exploratory borderline range surrounding the QFT-Plus positivity threshold and assessed the influence of the QFT-Plus upper quantification limit on quantitative comparisons.

## Materials and methods

Samples were obtained from patients referred for tuberculosis infection testing between June and October 2025 at the Microbiology Laboratory of Hospital Universitario Virgen de las Nieves. During the study period, 245 consecutive QuantiFERON tube samples were assessed for eligibility. A total of 19 samples were excluded according to the predefined exclusion criteria, which included age under 18 years, excess blood volume in the QFT tubes, duplicate samples from the same patient, unknown time from blood collection to receipt at the laboratory, and discrepancies between tube identification and request-form data. The final study sample therefore comprised 226 QuantiFERON tube samples from 226 unique individuals, with no repeated samples from the same patient (Figure 1). The demographic characteristics of the study cohort are summarized in Table 1, including the origin of the requests and the distribution of patients by sex and age groups. The sample size was based on consecutive eligible samples processed during the predefined study period for which paired results from both assays were available. No prospective formal sample-size calculation was performed. No participants were excluded due to clinical indications, underlying diseases, immunosuppressive treatments, or epidemiological risks of tuberculosis infection.

**Figure 1:**
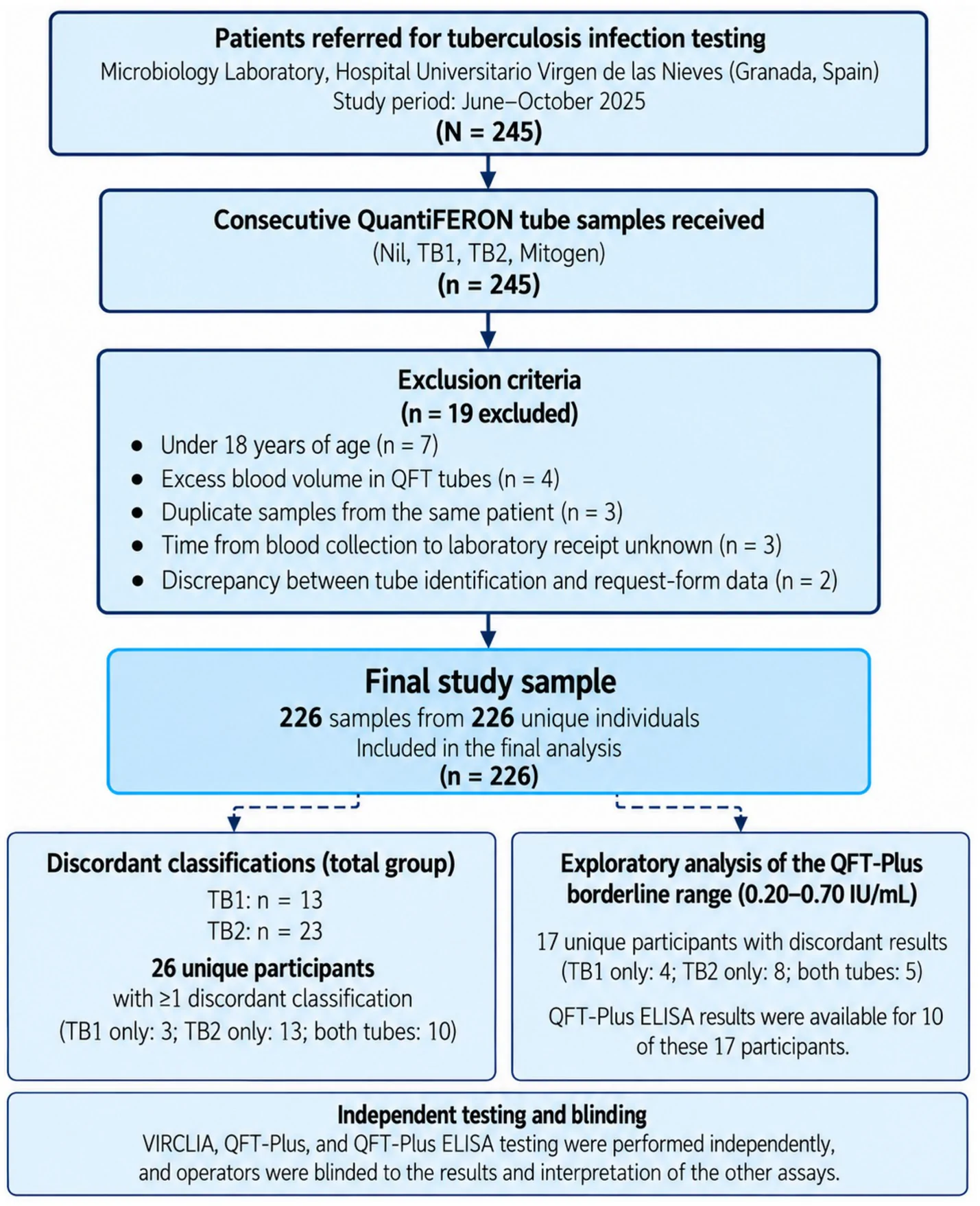
Participant flow, eligibility criteria, and study workflow. The diagram summarizes the source population, consecutive samples received, exclusions and reasons for exclusion, final study sample, and procedures for independent testing, blinding, and assessment of discordant results. Discordant classifications are shown separately for TB1 and TB2, along with the number of unique participants with at least one discordant classification. The exploratory analysis within the predefined borderline range (0.20–0.70 IU/mL) and the availability of QFT-Plus ELISA results for discordant samples within this range are also shown. QFT-Plus, QuantiFERON-TB Gold Plus; ELISA, enzyme-linked immunosorbent assay.

**Table 1:** Demographic characteristics of the study population.

Recruitment source/department	*N*	Female, *n* (%)	Mean age, years (range)
Correctional facility	74	14 (18.9)	42 (18–76)
Dermatology	51	28 (54.9)	38 (18–57)
Rheumatology	55	39 (70.9)	54 (21–83)
Other departments^*^	46	22 (47.8)	49 (19–81)
Total^**^	226	103 (45.6)	45 (18–83)

^*^Other departments included Internal Medicine, Infectious Diseases, and Hematology. ^**^Values are presented as *n* (%) for sex and mean age (range) in years.

In all samples, the LIAISON® QuantiFERON®-TB Gold Plus and the TB-IGRA VIRCLIA® Monotest were performed following the manufacturers’ recommendations. Within the exploratory borderline analysis, discordant samples for which ELISA results were available were further analyzed using the QuantiFERON®-TB Gold Plus ELISA kit following the manufacturer’s instructions. In both assays, the samples were processed using QuantiFERON®-TB Gold Plus blood collection tubes: Nil, Mitogen, TB1, and TB2. The stimulating antigens were ESAT-6 and CFP-10 (TB1: long peptides; TB2: short peptides). The total processing time was 16–24 h for antigen stimulation at 37 °C and 15 min for sample preparation (centrifugation).

In the present study, the TB-IGRA VIRCLIA® Monotest and QFT-Plus used plasma obtained from the same QFT-Plus blood collection and stimulation tubes (Nil, Mitogen, TB1, and TB2). Therefore, the comparative evaluation focused primarily on the IFN-γ detection and quantification methods rather than on differences in the antigen stimulation system.

The VIRCLIA Monotest and the QFT-Plus CLIA were performed independently, with operators blinded to the results of the other assay. QFT-Plus ELISA was performed only on a subset of discordant samples within the borderline range for which sufficient residual sample volume was available. ELISA testing could not be performed for the remaining samples because of insufficient residual sample volume. Therefore, ELISA operators were aware of the discordance but blinded to the specific results of the other assays. The participant selection process, eligibility criteria, and overall study workflow are summarized in Figure 1.

### TB-IGRA VIRCLIA® Monotest (Vircell)

In the TB-IGRA VIRCLIA® Monotest, interferon-gamma (IFN-γ) was determined using an automated sandwich chemiluminescent immunoassay. The assay was performed on an automated analyzer following a single incubation step of 40 min at 37 °C, during which the sample and conjugate were incubated simultaneously. Subsequently, the chemiluminescent substrate was added, followed by a second incubation of 10 min at 37 °C prior to signal measurement. The system employs a specific monoclonal antibody immobilized on a solid phase as the capture element and a second anti-IFN-γ monoclonal antibody conjugated to horseradish peroxidase (HRP) as the detection element. Patient samples were diluted to a 1:2 ratio with the conjugate before being dispensed into the reaction system. After substrate addition, the signal generated by the enzymatic reaction was measured in relative light units (RLUs). For each patient, four sample tubes were analyzed: Nil tube (negative control), TB1 and TB2 tubes (antigen-stimulated tubes), and the Mitogen tube (positive control).

The results were initially expressed in RLUs. To calculate the specific response, the difference between the TB1 and TB2 values and the Nil value was determined (TB1–Nil and TB2–Nil). These differences were interpolated against a standard curve generated using the international calibrator National Institute for Biological Standards and Control (NIBSC) 87/586, allowing conversion of the chemiluminescent signal into international units per milliliter (IU/mL). The final results for each patient were expressed as IU/mL of IFN-γ. The assay cutoff value was established at 0.35 IU/mL for both TB1 and TB2 (after subtraction of the Nil value). The results ≥0.35 IU/mL were considered positive. The test was classified as indeterminate when the Nil tube yielded a positive result (elevated IFN-γ concentration in IU/mL) and/or when the Mitogen tube yielded a negative result (insufficient IFN-γ response in IU/mL).

For an invalid Negative (N) control tube, the cutoff value was defined as ≥8 IU/mL. A Positive (P) control tube was considered invalid when the N tube value was ≤8 IU/mL and the mitogen value was ≤0.5 IU/mL. These criteria were used to define invalid results according to the quantitative values obtained for each tube.

### QuantiFERON®-TB Gold Plus CLIA (Qiagen)

The QuantiFERON®-TB Gold Plus CLIA (Qiagen) was performed using an automated chemiluminescent immunoassay platform. IFN-γ quantification was carried out using monoclonal capture and enzyme-labeled detection antibodies, with results expressed in IU/mL after interpolation against a calibration curve.

QFT-Plus results were interpreted following the manufacturer’s criteria: A patient was classified as positive when either TB1–Nil or TB2–Nil was ≥0.35 IU/mL and ≥25% of the Nil value, provided that Nil was ≤8.0 IU/mL; negative when neither antigen response met these criteria and Mitogen–Nil was ≥0.50 IU/mL; and indeterminate when Nil was >8.0 IU/mL or Mitogen–Nil was <0.50 IU/mL in the absence of a positive antigen response.

### QuantiFERON®-TB Gold Plus ELISA (Qiagen)

The QuantiFERON®-TB Gold Plus ELISA (Qiagen) is based on a solid-phase enzyme-linked immunosorbent assay (ELISA) employing a monoclonal capture antibody specific for IFN-γ and an enzyme-conjugated detection antibody. Briefly, the plasma samples were diluted to a 1:2 ratio with the conjugate and incubated for 2 h at room temperature to allow the binding of IFN-γ to the solid-phase–bound capture antibodies and subsequent interaction with the enzyme-conjugated detection antibody. After the incubation step, wells were washed to remove the unbound material. A chromogenic substrate was then added and incubated for 30 min at room temperature. The enzymatic reaction was stopped according to the manufacturer’s protocol, and optical density (OD) was measured using a microplate reader. Each run included four points of a standard curve. Optical density values were converted to international units per milliliter (IU/mL) by interpolation against the standard curve, and results were expressed as IU/mL.

For this assay, the positivity threshold was defined as 0.35 IU/mL for both TB1 and TB2 after correction by subtracting the Nil value. Responses equal to or greater than 0.35 IU/mL were interpreted as positive. An indeterminate result was assigned when the Nil control showed an elevated IFN-γ concentration (positive result in IU/mL) and/or when the Mitogen control failed to demonstrate an adequate IFN-γ response (negative result in IU/mL).

Table 2 summarizes the main operational characteristics of the three interferon-gamma quantification methods evaluated in this study, based on published evaluations and manufacturers’ technical documentation, highlighting differences in automation, throughput, workflow flexibility, and hands-on requirements (De Maertelaere et al., 2020; Fernández-Huerta et al., 2021; Vircell, 2026; DiaSorin, n.d.; Qiagen, 2021).

**Table 2:** Advantages and disadvantages of the evaluated methods for interferon-gamma quantification.

Technique	Advantages	Disadvantages
TB-IGRA VIRCLIA® Monotest	Automated chemiluminescent testing. Minimal manual handling. Single-sample monotest format. Does not require sample accumulation for batch processing. Flexible processing of low or irregular sample volumes. Allows on-demand processing of individual samples (Vircell, 2026; Calero et al., 2022)^*^.	Requires a dedicated automated platform. Lower maximum throughput than dedicated high-throughput CLIA platforms. Published independent experience with the TB-specific VIRCLIA® IGRA remains limited compared to the extensively evaluated QFT-Plus methodology (Vircell, 2026).
LIAISON® QFT-Plus CLIA	Fully automated IFN-γ measurement. Minimal hands-on laboratory time. Continuous-load/random-access operation. Short analytical turnaround time (<1 h). High throughput and suitability for high-volume laboratories (Fernández-Huerta et al., 2021; Kadkhoda et al., 2020; Buron and Banaei, 2023).	Requires a dedicated LIAISON® automated analyzer. Quantitative IFN-γ measurements may differ from conventional QFT-Plus ELISA, particularly for results close to the positivity threshold. Results close to the cutoff may therefore require cautious interpretation (Fernández-Huerta et al., 2021; Kadkhoda et al., 2020; Buron and Banaei, 2023).
QIAGEN QFT-Plus	Widely established and extensively evaluated IGRA methodology. Included among internationally recommended tests for tuberculosis infection. Can be performed using conventional ELISA laboratory equipment. Extensive published clinical and analytical experience (World Health Organization, 2025; Qiagen, 2019; Oh et al., 2021).	Longer analytical processing time than fully automated CLIA. Greater manual handling and pipetting requirements. Lower degree of automation than dedicated CLIA platforms. Predominantly batch-based microplate workflow. Less convenient for immediate processing of individual samples (Qiagen, 2019; Fernández-Huerta et al., 2021; Kadkhoda et al., 2020).

CLIA, chemiluminescence immunoassay; ELISA, enzyme-linked immunosorbent assay; IFN-γ, interferon gamma; IGRA, interferon-gamma release assay; QFT-Plus, QuantiFERON-TB Gold Plus. ^*^Reference (Calero et al., 2022) supports only the general operational characteristics of the VIRCLIA Monotest format.

### Assay kits and manufacturer involvement

The TB-IGRA VIRCLIA® Monotest, QFT-Plus CLIA, and QFT-Plus ELISA kits used in this study were purchased by the institution as part of routine laboratory practice and were not supplied free of charge by the respective manufacturers, who had no role in the study design, data collection, analysis, interpretation, or manuscript preparation.

### Statistical analysis

Qualitative agreement between QuantiFERON®-TB Gold Plus (QFT-Plus) and the TB-IGRA VIRCLIA® Monotest was assessed using the overall percent agreement, positive percent agreement (PPA), and negative percent agreement (NPA), with QFT-Plus as the comparator method. The 95% confidence intervals (CIs) for PPA and NPA were calculated using the Wilson score method. Agreement beyond chance was assessed using Cohen’s kappa (*κ*) coefficient, with corresponding 95% CIs. Qualitative agreement and Cohen’s *κ* were calculated for the overall patient-level classification and separately for TB1 and TB2 responses.

Quantitative agreement between QFT-Plus and TB-IGRA VIRCLIA® Monotest IFN-γ measurements was evaluated separately for TB1 and TB2. Deming regression was used to account for measurement errors in both methods, assuming an error-variance ratio of 1. Regression slopes and intercepts were reported with their 95% CIs. The Bland–Altman analysis was additionally performed by calculating the difference between paired measurements, specifically by subtracting the QFT-Plus results from the VIRCLIA results. The mean differences (bias) and 95% limits of agreement (LoA), calculated as the mean difference ±1.96 standard deviations of the paired differences, were reported.

In the study dataset, QFT-Plus measurements reached an upper quantification limit of 10 IU/mL, whereas VIRCLIA measurements exceeded this concentration, sensitivity analyses were performed to evaluate the influence of these upper-limit observations on the quantitative method comparison. Deming regression and Bland–Altman analyses were therefore repeated after excluding observations with QFT-Plus values of 10 IU/mL, separately for TB1 and TB2.

An exploratory borderline analysis was performed using an interval of 0.20–0.70 IU/mL around the 0.35 IU/mL positivity threshold. A sample was included in the TB1 or TB2 borderline analysis when at least one measurement from either assay for the corresponding tube fell within this interval. The 0.20–0.70 IU/mL interval was derived from previously published QFT-Plus literature and has not been independently validated for the TB-IGRA VIRCLIA® Monotest (Bisognin et al., 2020). Overall agreement and Cohen’s *κ* with 95% CIs were calculated separately within and outside the borderline range for TB1 and TB2. Discordant borderline samples were identified separately for TB1 and TB2, and an overlap between discordant samples in both tubes was assessed.

For discordant borderline samples with an available QFT-Plus ELISA result, the agreement of the qualitative classification obtained from QFT-Plus CLIA and the TB-IGRA VIRCLIA® Monotest compared to the ELISA results was assessed descriptively. Because QFT-Plus ELISA is not an independent clinical reference standard and was available for only a subset of discordant samples, this analysis was considered exploratory, and no inferential comparison between the methods was performed.

All statistical analyses were performed using R statistical software (version 4.5.3; R Foundation for Statistical Computing, Vienna, Austria). Statistical tests were two-sided, where applicable, and statistical significance was set at a *p*-value of <0.05.

### Ethical approval

The study was approved by the Ethics Committee of Hospital Universitario Virgen de las Nieves, Granada, Spain (approval code: 2504-1). All procedures were conducted in accordance with the principles of the Declaration of Helsinki and applicable institutional regulations.

## Results

A total of 226 paired samples were included in the comparative analysis. No indeterminate results were observed with QFT-Plus or the TB-IGRA VIRCLIA® Monotest. Regarding QFT-Plus control responses, the median Nil concentration was 0.087 IU/mL (interquartile range (IQR), 0.060–0.158; range, 0.018–7.510), while the median Mitogen concentration was 10.000 IU/mL (IQR, 10.000–10.000; range, 0.794–10.000). The median Mitogen–Nil response was 9.904 IU/mL (IQR, 9.605–9.935; range, 0.800–9.971). Notably, none of the 226 samples met the QFT-Plus control criteria for an indeterminate result: No sample showed a Nil concentration of >8.0 IU/mL or a Mitogen–Nil response of <0.50 IU/mL. Similarly, for the TB-IGRA VIRCLIA® Monotest, the median Negative Control concentration was 0.900 IU/mL (IQR, 0.501–1.905; range, 0.103–7.876), while the median Mitogen concentration was 163.978 IU/mL (IQR, 89.494–210.381; range, 1.205–375.165). The median Mitogen–Negative Control response was 162.271 IU/mL (IQR, 89.140–208.927; range, 0.949–374.733).

The overall qualitative agreement between the two assays was 92.9%, with a Cohen’s kappa coefficient of 0.85 (95% CI, 0.79–0.92). The positive percent agreement (PPA) was 90.1% (95% CI, 83.8–94.1), whereas the negative percent agreement (NPA) was 96.8% (95% CI, 91.1–98.9).

As shown in Table 3, agreement was higher for TB1 (94.25%; *κ* = 0.885; 95% CI, 0.824–0.945) than for TB2 (89.82%; *κ* = 0.796; 95% CI, 0.718–0.875).

**Table 3:** Qualitative agreement and quantitative method-comparison analyses between QFT-Plus and TB-IGRA VIRCLIA® Monotest for TB1 and TB2.

Analysis	TB1	TB2
Qualitative agreement		
All samples, *n*	226	226
Overall agreement, %	94.25	89.82
Cohen’s *κ* (95% CI)	0.885 (0.824–0.945)	0.796 (0.718–0.875)
Borderline analysis (0.20–0.70 IU/mL)		
Borderline samples, *n*	41	42
Agreement, %	78.05	69.05
Cohen’s *κ* (95% CI)	0.529 (0.289–0.769)	0.316 (0.067–0.564)
Outside borderline range		
Samples, *n*	185	184
Agreement, %	97.84	94.57
Cohen’s *κ* (95% CI)	0.957 (0.915–0.999)	0.891 (0.826–0.957)
Deming regression—all samples		
*n*	226	226
Intercept (95% CI)	−3.652 (−5.023 to −2.281)	−3.902 (−5.445 to −2.358)
Slope (95% CI)	3.085 (2.722–3.448)	3.146 (2.750–3.542)
Deming regression—QFT-Plus <10 IU/mL		
*n*	204	203
Intercept (95% CI)	−0.115 (−0.218 to −0.012)	−0.190 (−0.401 to 0.021)
Slope (95% CI)	0.811 (0.765–0.858)	0.926 (0.837–1.015)
Bland–Altman—all samples		
Mean bias, IU/mL	0.770	0.840
95% LoA, IU/mL	−10.528 to 12.069	−10.924 to 12.604
Bland–Altman—QFT-Plus <10 IU/mL		
*n*	204	203
Mean bias, IU/mL	−0.355	−0.288
95% LoA, IU/mL	−1.774 to 1.063	−2.527 to 1.950

Qualitative agreement was reported for the complete dataset, within the exploratory borderline range, and outside the borderline range. The borderline range was defined as 0.20–0.70 IU/mL when at least one measurement from either assay fell within this interval. This interval was derived based on the QFT-Plus literature and has not been independently validated for VIRCLIA. Deming regression and Bland–Altman analyses were performed for the complete dataset and in sensitivity analyses excluding observations at the QFT-Plus upper quantification limit of 10 IU/mL. Bland–Altman differences were calculated by subtracting QFT-Plus values from VIRCLIA values. CI, confidence interval; LoA, limits of agreement; QFT-Plus, QuantiFERON-TB Gold Plus.

The relationship between QFT-Plus and TB-IGRA VIRCLIA® Monotest quantitative measurements is shown in Figure 2. In the analysis including all 226 samples (Figures 2A,B), Deming regression showed slopes substantially greater than the slope of the line of identity for both TB1 and TB2. For TB1, the slope was 3.085 (95% CI, 2.722–3.448), with an intercept of −3.652 (95% CI, −5.023 to −2.281). For TB2, the slope was 3.146 (95% CI, 2.750–3.542), with an intercept of −3.902 (95% CI, −5.445 to −2.358). At higher IFN-γ concentrations, several QFT-Plus measurements reached the upper quantification limit of 10 IU/mL, whereas VIRCLIA measurements continued to increase, potentially influencing the Deming regression estimates.

**Figure 2:**
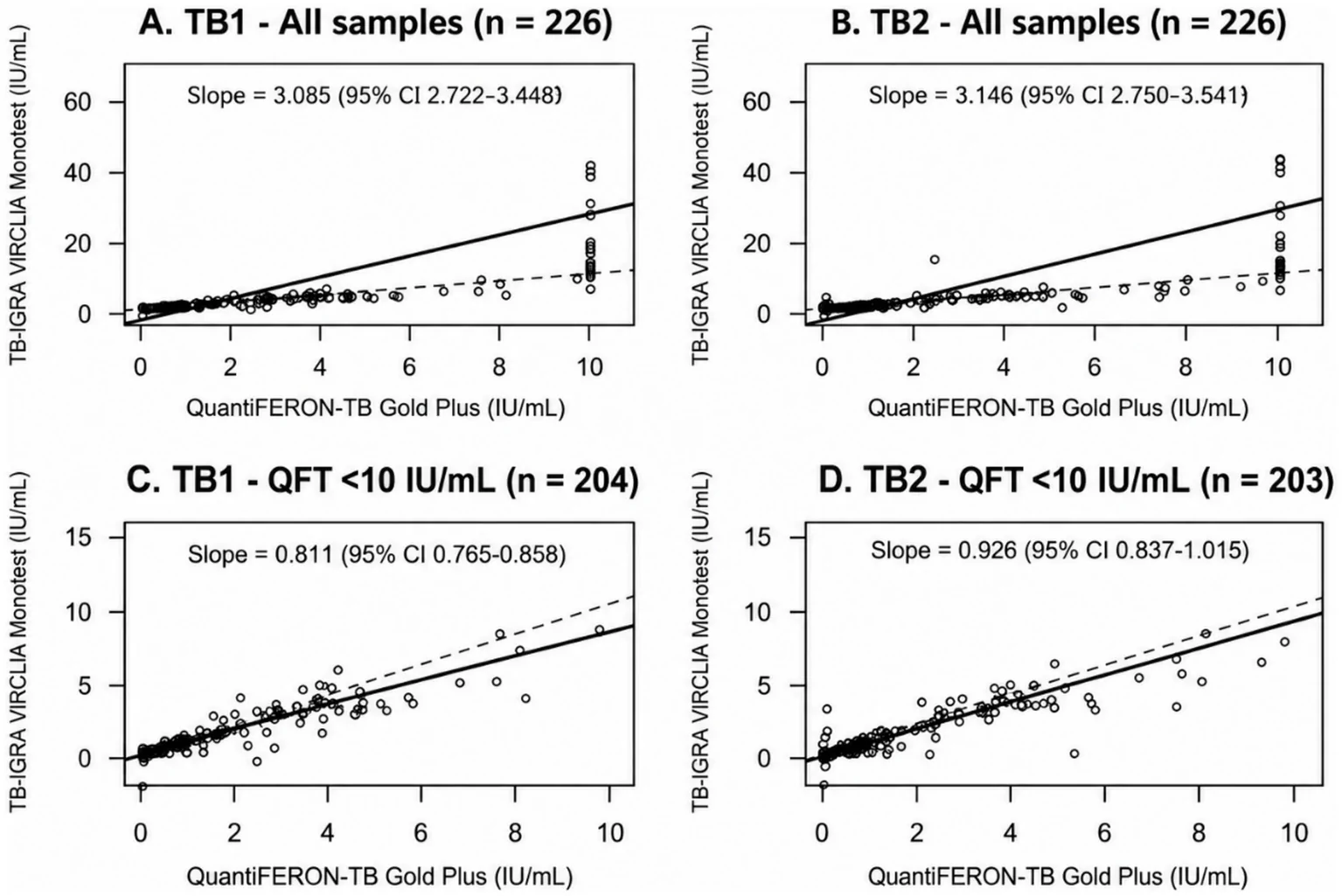
Deming regression analysis of quantitative interferon-gamma (IFN-γ) measurements obtained using QFT-Plus and TB-IGRA VIRCLIA® Monotest. Panels **A** and **B** show the analysis including all 226 samples for TB1 and TB2, respectively. Panels **C** and **D** show the corresponding sensitivity analyses after excluding observations at the QFT-Plus upper quantification limit of 10 IU/mL (TB1, *n* = 204; TB2, *n* = 203). The solid black line represents the Deming regression fit, and the dashed line represents the line of identity (*y* = *x*). The sensitivity analysis was performed to assess the influence of the QFT-Plus upper quantification limit on the regression estimates. Deming regression slopes and their 95% confidence intervals are displayed within each panel. QFT-Plus, QuantiFERON-TB Gold Plus.

To assess the influence of this upper measurement limit, a sensitivity analysis was performed after excluding observations with QFT-Plus values of 10 IU/mL (Figures 2C,D). A total of 22 observations were excluded for TB1, and 23 observations were excluded for TB2, leaving 204 and 203 samples, respectively. The Deming slope decreased to 0.811 (95% CI, 0.765–0.858) for TB1, with an intercept of −0.115 (95% CI, −0.218 to −0.012), and to 0.926 (95% CI, 0.837–1.015) for TB2, with an intercept of −0.190 (95% CI, −0.401 to 0.021). These findings indicate that the steep slopes observed in the complete dataset were substantially influenced by the observations at the QFT-Plus upper quantification limit.

The Bland–Altman analysis was consistent with the influence of the QFT-Plus upper quantification limit observed in the Deming regression (Figure 3). In the analysis including all 226 samples (Figures 3A,B), the mean difference (VIRCLIA–QFT-Plus) was 0.770 IU/mL for TB1, with 95% limits of agreement (LoA) from −10.528 to 12.069 IU/mL, and 0.840 IU/mL for TB2, with 95% LoA from −10.924 to 12.604 IU/mL. After excluding the observations at the QFT-Plus upper quantification limit of 10 IU/mL (Figures 3C,D), the mean differences decreased to −0.355 IU/mL for TB1 (95% LoA, −1.774 to 1.063 IU/mL; *n* = 204) and −0.288 IU/mL for TB2 (95% LoA, −2.527 to 1.950 IU/mL; *n* = 203). The markedly narrower limits of agreement in the sensitivity analysis indicate that observations at the QFT-Plus upper quantification limit substantially contributed to the dispersion observed in the complete dataset.

**Figure 3:**
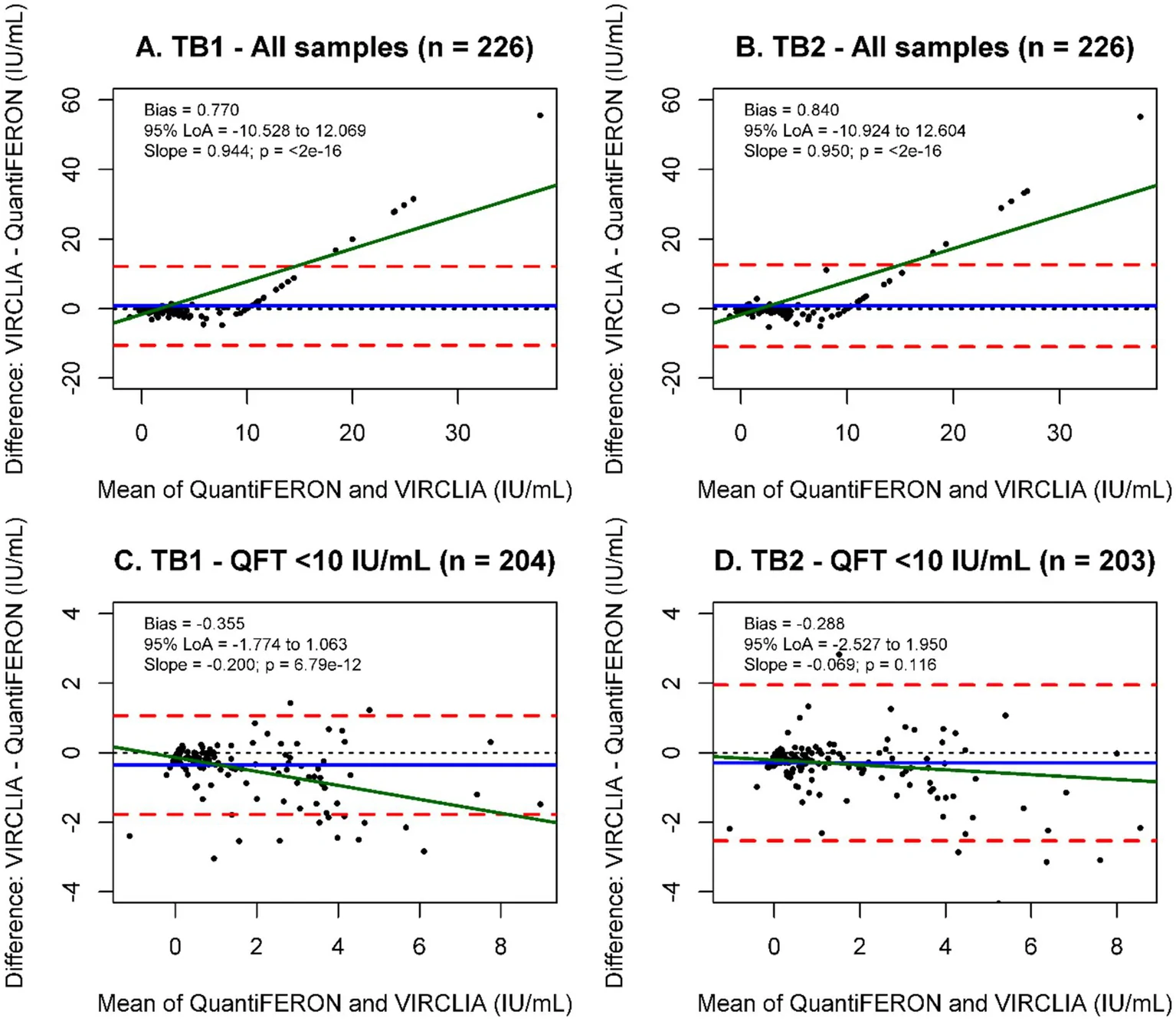
Bland–Altman analysis of quantitative IFN-γ measurements obtained using QFT-Plus and TB-IGRA VIRCLIA® Monotest. Panels **A** and **B** show the analysis including all 226 samples for TB1 and TB2, respectively. Panels **C** and **D** show the corresponding sensitivity analyses after excluding observations at the QFT-Plus upper quantification limit of 10 IU/mL (TB1, *n* = 204; TB2, *n* = 203). Differences were calculated as VIRCLIA minus QFT-Plus. The solid blue line represents the mean difference (bias), the dashed red lines represent the 95% limits of agreement, and the dotted black line represents zero difference. The solid green line represents the linear regression of the differences against the mean IFN-γ concentration. The sensitivity analysis was performed to assess the influence of observations at the QFT-Plus upper quantification limit on the agreement between methods. QFT-Plus, QuantiFERON-TB Gold Plus.

The distribution of quantitative IFN-γ measurements obtained using QFT-Plus and the TB-IGRA VIRCLIA® Monotest is shown in Figure 4. Panel A displays the distribution across the full measurement range on a logarithmic scale, whereas Panel B provides a detailed view of values near the 0.35 IU/mL positivity threshold.

**Figure 4:**
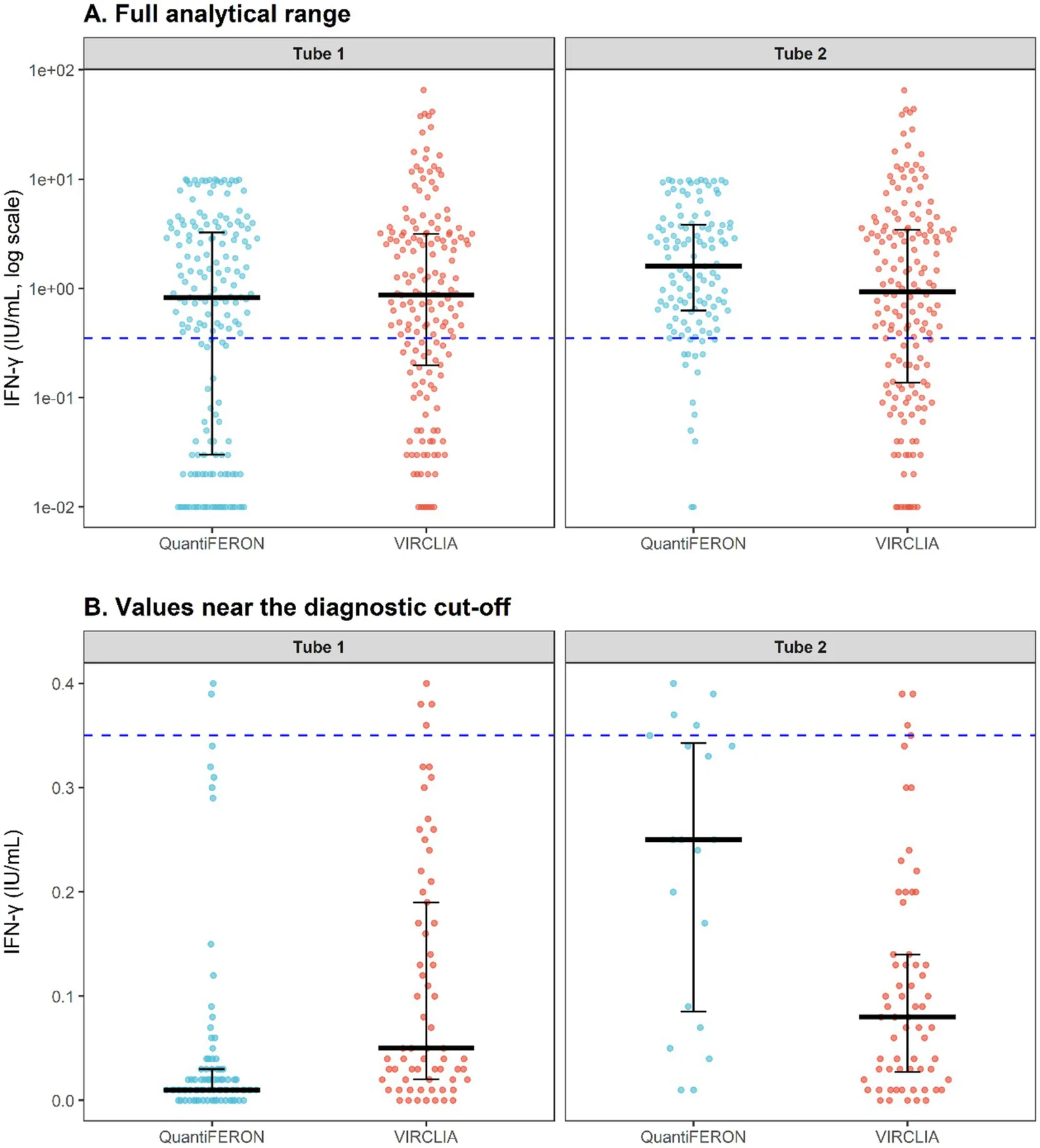
Distribution of IFN-γ measurements across the full measurement range and near the positivity threshold for QFT-Plus and TB-IGRA VIRCLIA® Monotest. Panel **A** shows the distribution of IFN-γ measurements for TB1 and TB2 across the full measurement range using a logarithmic scale. Panel **B** provides a detailed view of measurements near the 0.35 IU/mL positivity threshold. Each point represents an individual measurement and is displayed using quasirandom jitter to improve visualization of data density. Black horizontal bars indicate the median, and error bars represent the interquartile range. The blue dashed line indicates the 0.35 IU/mL positivity threshold. Values ≤ 0 were excluded from Panel A for visualization purposes because of the logarithmic scale but were retained in the statistical analyses. QFT-Plus, QuantiFERON-TB Gold Plus.

When restricting the analysis to the exploratory borderline range (0.20–0.70 IU/mL), 41 samples were classified as borderline for TB1, and 42 samples were classified as borderline for TB2. Within this range, agreement was 78.05% for TB1 (*κ* = 0.529; 95% CI, 0.289–0.769) and 69.05% for TB2 (*κ* = 0.316; 95% CI, 0.067–0.564). In contrast, outside the borderline range, agreement increased to 97.84% for TB1 (*κ* = 0.957; 95% CI, 0.915–0.999) and 94.57% for TB2 (*κ* = 0.891; 95% CI, 0.826–0.957).

Discordant results were predominantly observed within the borderline range (Figure 5). Among borderline samples, 9 were identified as discordant for TB1 and 13 for TB2. Five samples were discordant for both TB1 and TB2, whereas 4 were discordant for TB1 only and 8 for TB2 only, corresponding to 17 unique discordant samples within the borderline range. The five samples showing discordance in both tubes are individually highlighted in Figure 5.

**Figure 5:**
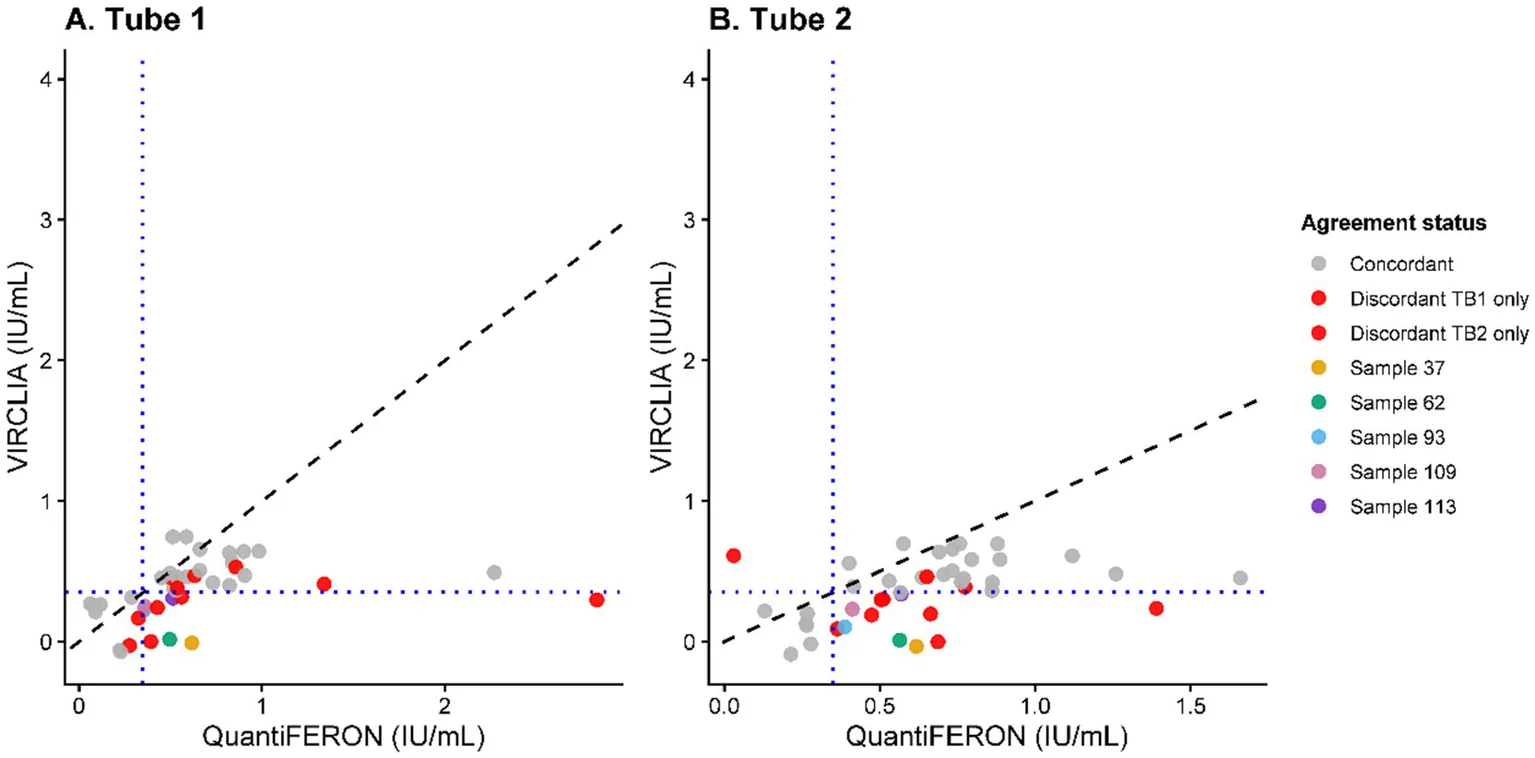
Agreement between QFT-Plus and TB-IGRA VIRCLIA® Monotest within the borderline IFN-γ range. Panels **A** and **B** show QFT-Plus versus VIRCLIA quantitative IFN-γ measurements for TB1 (*n* = 41) and TB2 (*n* = 42), respectively, restricted to samples in which at least one measurement from either assay was within the exploratory borderline range of 0.20–0.70 IU/mL. The dashed diagonal line represents the line of identity (*y* = *x*), and the dotted horizontal and vertical lines indicate the 0.35 IU/mL positivity threshold. Concordant samples are shown in gray, whereas discordant samples are highlighted. Samples discordant exclusively for TB1 or TB2 are shown in red, and the five samples discordant for both TB1 and TB2 are individually color-coded using the same color in both panels. The 0.20–0.70 IU/mL interval was used as an exploratory QFT-Plus-derived borderline range and has not been independently validated for VIRCLIA. QFT-Plus, QuantiFERON-TB Gold Plus.

Among the 17 unique discordant samples identified within the borderline analysis, 10 had an available QFT-Plus ELISA result. QFT-Plus and VIRCLIA each agreed with the ELISA in 7 of 10 cases (70%). Both assays agreed with the ELISA in four cases, whereas QFT-Plus alone agreed with the ELISA in three cases and VIRCLIA alone in three cases. Due to the limited number of samples and the fact that QFT-Plus ELISA does not constitute an independent clinical reference standard, this exploratory comparison was considered inconclusive, failing to determine which assay provided the correct classification.

Overall, the qualitative agreement between QFT-Plus and the TB-IGRA VIRCLIA® Monotest was high, but the agreement decreased substantially within the exploratory borderline range, particularly for TB2. Outside the borderline range, qualitative agreement was substantially higher for both TB1 and TB2. Quantitative method-comparison analyses were strongly influenced by observations at the QFT-Plus upper quantification limit, as demonstrated by the Deming regression and Bland–Altman sensitivity analyses.

## Discussion

In the present study, the TB-IGRA VIRCLIA® Monotest showed high qualitative agreement with QFT-Plus. The overall agreement was 92.9%, with a Cohen’s kappa coefficient of 0.85 (95% CI, 0.79–0.92), a positive percent agreement (PPA) of 90.1% (95% CI, 83.8–94.1), and a negative percent agreement (NPA) of 96.8% (95% CI, 91.1–98.9). Agreement was high when TB1 and TB2 responses were evaluated separately, although it was lower for TB2 than for TB1. These findings are broadly consistent with previous method-comparison studies of recently introduced IGRA platforms, which have also reported substantial agreement with established assays (Céspedes et al., 2025; Crudu et al., 2025; Turbawaty et al., 2025).

Quantitative analyses showed substantial differences between the complete dataset and the analysis excluding observations at the QFT-Plus upper quantification limit. In the complete dataset, Deming regression yielded slopes of 3.085 (95% CI, 2.722–3.448) for TB1 and 3.146 (95% CI, 2.750–3.542) for TB2. However, after excluding observations at the QFT-Plus upper quantification limit of 10 IU/mL, the slopes decreased to 0.811 (95% CI, 0.765–0.858) for TB1 and 0.926 (95% CI, 0.837–1.015) for TB2. The Bland–Altman analysis showed a similar pattern, with markedly narrower limits of agreement after excluding these observations. These findings indicate that the apparent quantitative divergence observed in the complete dataset, particularly at higher IFN-γ concentrations, was substantially influenced by the upper quantification limit of QFT-Plus. Therefore, quantitative differences between the two platforms at the upper measurement range should be interpreted cautiously.

A particularly relevant finding was the marked reduction in agreement within the exploratory borderline range (0.20–0.70 IU/mL) around the 0.35 IU/mL positivity threshold. For TB1, agreement decreased from 97.84% (*κ* = 0.957; 95% CI, 0.915–0.999) outside the borderline range to 78.05% (*κ* = 0.529; 95% CI, 0.289–0.769) within this range. The reduction was more pronounced for TB2, with agreement decreasing from 94.57% (*κ* = 0.891; 95% CI, 0.826–0.957) outside the borderline range to 69.05% (*κ* = 0.316; 95% CI, 0.067–0.564) within the borderline range. These findings indicate that while there was a high overall agreement between the two methods, it was not uniform across the measurement range and was substantially lower for results close to the positivity threshold. This pattern is consistent with previous studies reporting greater variability and discordance around the 0.35 IU/mL threshold (Fernández-Huerta et al., 2021).

In the current context of increasing diversification of IGRA technologies, several assays based on ELISA, lateral-flow immunochromatography, and automated chemiluminescent detection have been developed for measuring IFN-γ. The TB-IGRA VIRCLIA® Monotest incorporates automated chemiluminescent IFN-γ detection in a monotest format, enabling individual samples to be processed without conventional batch testing. These characteristics distinguish its analytical workflow from conventional ELISA-based methods; however, the present study did not formally evaluate hands-on time, turnaround time, reagent consumption, operator workload, or costs. Therefore, the operational and economic implications of these technical characteristics require dedicated evaluation.

The WHO has highlighted the importance of expanding access to tuberculosis infection testing. The availability of additional tuberculosis infection testing platforms may contribute to increasing flexibility across different laboratory settings [World Health Organization (WHO), 2022].

An updated WHO-supported systematic review of newly commercialized IGRAs identified several emerging platforms, including LIAISON QFT-Plus, VIDAS TB-IGRA, STANDARD TB-Feron, Wantai CLIA, and AdvanSure TB-IGRA, which generally showed high agreement with established WHO-endorsed assays; however, the overall certainty of evidence remained limited by small study sizes and methodological heterogeneity. In this context, independent evaluations of additional platforms, such as the TB-IGRA VIRCLIA® Monotest, are important to further characterize their analytical characteristics and agreement with established methods [World Health Organization (WHO), 2022].

No indeterminate results were observed with either assay in the present cohort. This precluded a direct comparison of the performance of QFT-Plus and the TB-IGRA VIRCLIA® Monotest in indeterminate samples. Further studies, specifically which includes patients with indeterminate IGRA results, are needed to determine whether differences exist between the platforms in their capability to classify these samples.

The reduced agreement observed within the exploratory borderline range is particularly relevant in light of recent evidence comparing different IFN-γ detection platforms. A recent systematic review and meta-analysis comparing QFT-Plus CLIA with ELISA found substantial overall agreement but also platform-dependent discordance and considerable heterogeneity, emphasizing that analytical agreement does not necessarily imply clinical equivalence (Bongomin et al., 2026). Furthermore, a large real-world study including 22,392 IGRAs reported an 87.8% relative increase in borderline-positive results after the implementation of the QFT-Plus CLIA, whereas non-borderline positivity did not significantly change; importantly, some of these changes in classification resulted in modifications to clinical management (Wilber et al., 2026). These findings, together with the substantially lower agreement observed within the exploratory borderline range in our study, support cautious interpretation of results close to the positivity threshold and suggest that repeat or confirmatory testing may be appropriate when classification could influence clinical management.

The 0.20–0.70 IU/mL interval used in the present study was based on existing data from QFT-Plus (Bisognin et al., 2020) and has not been independently validated for the TB-IGRA VIRCLIA® Monotest. Therefore, the borderline analysis should be considered exploratory, and the present findings should not be interpreted as establishing a borderline range for VIRCLIA. Further studies are required to determine whether a platform-specific borderline range is appropriate for VIRCLIA.

Discordant classifications were more frequently observed within the exploratory borderline range. Although analytical and biological variability around the positivity threshold could contribute to such discordance, the present study was not designed to establish its underlying cause. Among the 17 unique discordant samples identified within the borderline analysis, QFT-Plus ELISA results were available for only 10 samples. This exploratory comparison could not determine which assay provided the correct classification because of the limited number of samples and because the QFT-Plus ELISA does not constitute an independent clinical reference standard.

This study has several limitations. First, it was conducted at a single center with a moderate sample size, which may limit the generalizability of the findings to other populations and laboratory settings. Second, both the TB-IGRA VIRCLIA® Monotest and QFT-Plus were evaluated using the same QFT-Plus blood collection and antigen stimulation tubes (Nil, Mitogen, TB1, and TB2). Consequently, the present study primarily compares the subsequent IFN-γ detection and quantification platforms rather than two completely independent IGRA systems, and the observed agreement should be interpreted within this shared antigen-stimulation framework. Third, the study lacked an independent clinical reference standard for tuberculosis infection. Therefore, the present findings reflect agreement between the TB-IGRA VIRCLIA® Monotest and QFT-Plus and should not be interpreted as estimates of diagnostic sensitivity, specificity, or accuracy. Although QFT-Plus was used as the comparator method, discordance with QFT-Plus does not necessarily indicate an incorrect VIRCLIA result, and the present study cannot determine which assay provides the correct classification in discordant cases. Fourth, the 0.20–0.70 IU/mL borderline interval was derived from published experience with QFT-Plus and has not been independently validated for the TB-IGRA VIRCLIA® Monotest; therefore, analyses within this range should be considered exploratory. Fifth, the additional ELISA comparison was available for only 10 of the 17 unique discordant samples identified within the borderline analysis, and because the QFT-Plus ELISA does not constitute an independent clinical reference standard, this exploratory analysis could not resolve the discordant classifications. Sixth, detailed information on immune status was not systematically collected; therefore, subgroup analyses according to immunosuppression could not be performed. This limitation is particularly relevant when interpreting the absence of indeterminate results, and the applicability of these findings to immunocompromised populations should be considered with caution. Finally, the study did not assess analytical reproducibility, longitudinal variability, or operational and economic outcomes such as hands-on time, turnaround time, reagent consumption, operator workload, and costs. These aspects should be addressed in future studies to further characterize the analytical and clinical utility of the platform.

In conclusion, the TB-IGRA VIRCLIA® Monotest showed high overall qualitative agreement with QFT-Plus in this cohort, particularly for the results outside the exploratory borderline range. Agreement was lower near the positivity threshold, especially for TB2, supporting cautious interpretation of the results in this range. Quantitative comparisons at higher IFN-γ concentrations were substantially influenced by the upper quantification limit of QFT-Plus. In addition, its automated single-sample Monotest format may provide operational flexibility by allowing individual samples to be processed without the need for batch accumulation, which could be particularly useful in laboratories with low or irregular IGRA workloads. Further studies incorporating independent clinical reference standards, reproducibility assessments, and clinically characterized populations are needed to establish the diagnostic performance and clinical utility of the TB-IGRA VIRCLIA® Monotest.

## Data Availability

The raw data supporting the conclusions of this article will be made available by the authors, without undue reservation.
